# Treatment of sudden sensorineural hearing loss with tinnitus with Fu subcutaneous needling: A case report

**DOI:** 10.1097/MD.0000000000041100

**Published:** 2024-12-20

**Authors:** Jiarui Zhang, Jin Lu, Yue Wang, Xinghao Ding

**Affiliations:** a Nanjing University of Chinese Medicine, Nanjing, China; b Nanjing Hospital Of Chinese Medicine Affiliated to Nanjing University of Chinese Medicine, Nanjing, China; c The Community Health Service Center of Jingan in Qixia District, Nanjing, China.

**Keywords:** Fu subcutaneous needling, sudden sensorineural hearing loss, tinnitus

## Abstract

**Rationale:**

Fu’s subcutaneous needling (FSN) is a special acupuncture method that uses FSN to sweep the subcutaneous tissue around or adjacent to the limbs to disperse the pain. Sudden sensorineural hearing loss (SSHL) is a kind of otological emergency with sudden onset within 72 hours, with unilateral hearing loss, and hearing loss of ≥20 dB in 2 connected frequencies, and most of the patients have no obvious triggers. Most patients with SSHL are accompanied by tinnitus, vertigo, and nausea and vomiting.

**Patient concerns:**

The patient suffered from hearing loss with tinnitus. Although received relevant treatment, the hearing loss and tinnitus did not improve significantly.

**Diagnoses:**

SSHL with tinnitus.

**Interventions:**

FSN treatment. A point 5 cm lateral to the point of tension and stiffness of the left trapezius muscle was selected on the affected back as the needle insertion point, and the condition of the neck muscles was improved through the FSN sweeping movement with neck reperfusion activities. The patient received treatment twice a week, and 4 weeks of treatment as a phase. At the end of each phase, a pure tone threshold audiometry, tinnitus evaluation scale Tinnitus Handicap Inventory assessment, and neck muscle palpation were performed.

**Outcomes:**

The patient’s hearing improved and the Tinnitus Handicap Inventory score decreased.

**Lessons:**

FSN has a good therapeutic effect on SSHL with tinnitus.

## 1. Introduction

Sudden sensorineural hearing loss (SSHL) is a kind of otological emergency with sudden onset within 72 hours, with unilateral hearing loss, and hearing loss of ≥20 dB in 2 connected frequencies, and most of the patients have no obvious triggers.. As we all know, there is currently no specific treatment for sudden deafness. Currently, modern medicine usually uses drug therapy, but long-term use of drugs can damage human liver and kidney function.

In this case report, we introduce a new alternative medicine called Fu subcutaneous needling (FSN). We find that it can improve the patient’s hearing and reduce the loudness and duration of tinnitus by FSN. This is the first paper to report on the treatment of sudden deafness with FSN. Therefore, this paper is innovative. We think this can expand the scope of FSN treatment and provide new perspectives on the treatment of SSHL.

SSHL is a disease in which hearing loss of 20 dB or more in at least 2 adjacent frequencies occurs suddenly within 3 days or less. The disease can occur at any age, mainly in people aged 43 to 53 years old, and most of them have unilateral onset, while the bilateral incidence rate is only 1.7% to 4.9%.^[1]^ Long-term hearing loss ultimately affects the patient’s physical and mental condition, psychological health, and quality of life. SSHL is often accompanied by tinnitus, dizziness, nausea, and vomiting. Among them, tinnitus is closely related to SSHL, and about 10.6% of SSHL patients are accompanied by tinnitus.^[2]^

Currently, for patients with sudden deafness, it is usually recommended to go to the hospital quickly within 7 days of hearing loss to receive relevant treatments,^[3]^ such as vasodilators to improve the inner ear circulation, glucocorticoids anti-inflammatory, neurotrophic agents to repair the damaged neurons and so on.^[4]^ However, the effect of drugs on the treatment of SSHL is not stable, and long-term use of drugs is often accompanied by side effects, causing damage to cardiovascular, liver, and kidney function.^[5,6]^ Hyperbaric oxygen therapy is used in SSHL as a rescue therapy.^[7]^ One study described that patients who received hyperbaric oxygen combined with medication within 10 days of hearing loss had significantly higher efficacy than patients treated after 10 days.^[8,9]^ FSN is a special method of acupuncture that uses FSN to sweep the subcutaneous tissue around or adjacent to the limbs to disperse the pain.^[10]^ Although acupuncture is easy to perform and has few side effects, there are no relevant reports about the use of FSN in the treatment of SSHL.

In this case, we report a case of SSHL with tinnitus, in which hearing was gradually restored and tinnitus was relieved after 2 months of treatment with FSN. In order to provide new ideas for the treatment of SSHL with tinnitus.

## 2. Case information

The patient male,49 years old, presented to the Department of Acupuncture and Moxibustion with a 15-day history of hearing loss with tinnitus in the left ear. History of present illness: The patient complained that 15 days ago, he felt stuffiness in his left ear with cicadas-like tinnitus in the morning. On the way to the company, when listening to music with headphones on, it was obvious that the volume of the left ear was weaker than that of the right ear, and when driving, he felt that the volume of the horn of the vehicle behind him was significantly weaker. The patient was tested for Pure tone threshold audiometry (PTA) in the ENT department of our hospital, and the result of the examination was: 250Hz-500-1K-2K-4K-8K (90dB-90-90-90-90-90-100) (Fig. 2A). The diagnosis was “SSHL with tinnitus in the left ear.” The patient was hospitalized to have the relevant medication such as methylcobalamin, postauricular drug injection, corticosteroid therapy, and the hyperbaric oxygen therapy to improve the microcirculation. However, the patient felt that his treatment was not effective, and then he went to the Department of Acupuncture and Moxibustion to seek further treatment. As the long-term hearing loss with tinnitus was not relieved, he had emotional anxiety and decreased sleep quality. Past history: The patient had no previous history of tinnitus and no history of exogenous ear injury. Physical examination: DR of the atlantoaxial joint showed straightening of cervical curvature, calcification of the collateral ligament, and unequal distance between the cardinal vertebral odontoid process and the left and right atlantoaxial blocks. Brain MRI: brain MRI scan showed no significant abnormality. The patient had tension and stiffness in the left sternocleidomastoid, left trapezius, left scalene, and left supraspinatus muscles. Cervical range of motion was normal. Tinnitus Handicap Inventory (THI) score: 46. The diagnosis was left-sided SSHL with tinnitus.

**Figure 1.**
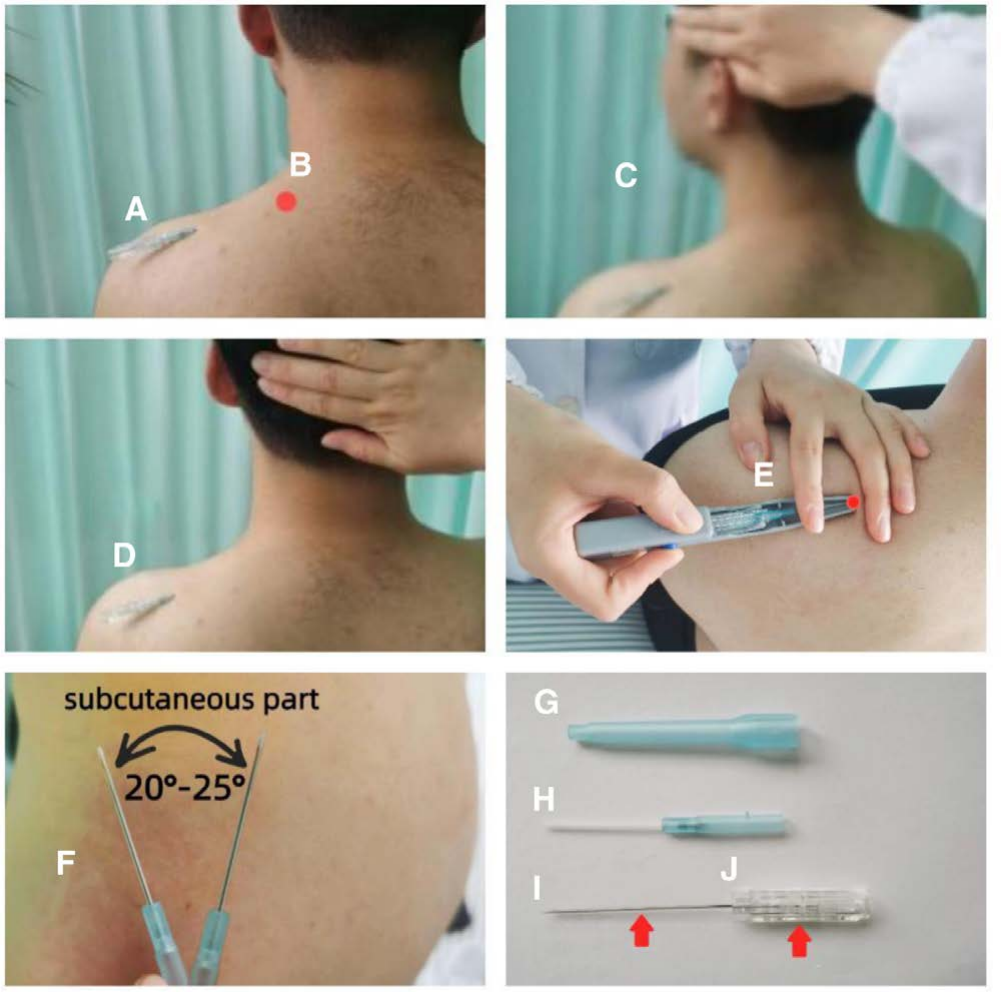
(A) The needle insertion point. (B) Muscle tension and stiffness points of the trapezius muscle. (C) Ipsilateral head-turning resistance. (D) Head-up resistance. (E) A needle inserting device. (F) Sweeping motion. (G) A needle cap. (H) The hose cannula. (I) Needle core. (J) The core holder.

**Figure 2.**
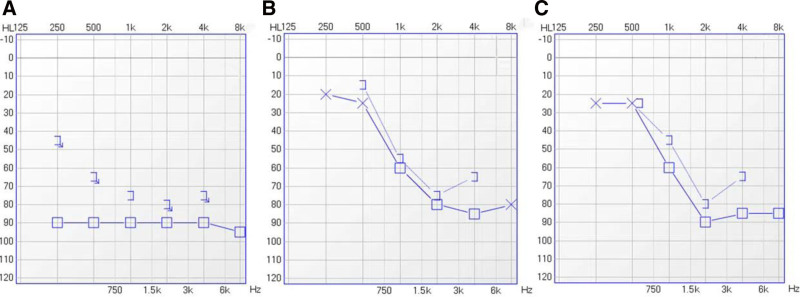
(A) Left ear pure tone threshold audiometry on October 12, 2023. (B) Left ear pure tone threshold audiometry on November 23, 2023. (C) Left ear pure tone threshold audiometry on December 27, 2023.

## 3. Therapeutic process

Considering the pretreatment examination of the cervical muscles and the results of audiometry, FSN was chosen as the treatment plan. FSN was designed and patented in China (Patent No. CN204932248U, Nanjing Paifu Medical Technology Co., Ltd, Nanjing, Jiangsu, China). FSN manipulation: A point 5cm lateral to the point of tension and stiffness (Fig. 1B) of the left trapezius muscle was selected on the affected back as the needle insertion point (Fig. 1A).Disinfected the needle insertion and the needle inserting device with 75% alcohol. Remove a needle cap (Fig. 1G). By using a needle inserting device (Fig. 1E) at a 15 to 20 angle to the skin, the FSN was inserted into the subcutaneous tissue. After the hose cannula (Fig. 1H) completely entered the subcutaneous layer, the core of the needle (Fig. 1I) was retracted into the hose cannula. Take the needle insertion as the fulcrum, the tip of the right thumb was fixed on the skin as the fulcrum, the core holder (Fig. 1J) was held by the right thumb and middle finger, and the index finger and ring finger exerted force to make the needle body do a see-saw-like sweeping motion (Fig. 1F) with the radian between 20 to 25 angle. The frequency of sweeping movement was about 100 times per minute. Duration of the sweeping movement was <1 minute. While sweeping, the patient was made to do the reperfusion activities (RA) of the Ipsilateral head-turning resistance (Fig. 1C) and the head-up resistance (Fig. 1D). Each RA lasted for about 5 to 10s. The patient relaxed for about 1 minute and then the RA was performed again. After thirty minutes, checked the stiff muscle, if it was not relieved, repeat the above treatment steps. When it was improved, removed the needle and press the entry point with a dry cotton ball for 2 minutes to prevent bleeding.

The patient received treatment twice a week, and 4 weeks of treatment as a phase. At the end of each phase, a PTA, tinnitus evaluation scale THI assessment, and neck muscle palpation were performed. At the end of the first phase of treatment, the patient’s low-frequency hearing was significantly improved (Fig. 2B), the THI score was 30, and the tinnitus symptoms were relieved significantly. The patient reported a significant decrease in stuffy sensation in the ear and the duration and loudness of the tinnitus. Neck muscle palpation: Tightness and stiffness of neck muscles improved significantly. At the end of the second phase of treatment, the patient’s high-frequency hearing improved, low-frequency and mid-frequency hearing was maintained well (Fig. 2C), tinnitus loudness and duration were relieved, and the THI score was 30. Neck muscle palpation: There is no obvious tension or stiffness in the neck muscles. The patient chose to end the treatment after complaining of hearing recovery, good sleep, and tinnitus that did not affect daily life. Four weeks after the end of treatment, we followed up with the patient by telephone, the patient reported that he still had slight tinnitus, but it did not affect his life, and the symptoms were similar to those at the end of treatment 4 weeks ago, without aggravation, and he had a THI score of 12. No adverse and unexpected events occurred to the patient during the treatment period.

## 4. Discussion

### 4.1. Pathogenesis

It believes that the etiology and pathogenesis of SSHL have not yet been clarified, and are mostly related to viral infections, local blood circulation disorders, and autoimmune dysfunction.^[11]^ Several studies have concluded that insufficient blood supply to the inner ear is closely related to SSHL, and microcirculatory disorders of the inner ear are also considered to be a key etiological factor of SSHL.^[12]^ The main source of blood supply to the inner ear is the labyrinthine artery, a branch of the vertebrobasilar artery. When the vertebrobasilar artery occurs vasospasm, vascular embolism, or poor blood circulation, resulting in poor blood flow of the labyrinthine artery and the blood supply to the inner ear is insufficient, SSHL can occur.^[13]^ Some studies have shown that the resistance index and blood flow velocity of the relevant artery on the affected side of patients with unilateral SSHL are significantly lower than those on the healthy side.^[14]^ In patients with SSHL and tinnitus, when hearing improves, tinnitus symptoms will also be relieved.^[15]^ Therefore, improving inner ear blood supply is very important in the treatment of SSHL with tinnitus.

### 4.2. FSN mechanism

The neck muscles cover the vertebrobasilar artery, and the contraction and diastole of the muscles are important factors affecting blood flow.^[16]^ Studies have shown that some patients with SSHL and tinnitus have high muscle tone on the affected side. Long-term muscle tension will affect the local tissue fluid and blood circulation.^[17]^ Relieving muscle stiffness can improve blood flow rates and muscle oxygen saturation.^[18]^ FSN mainly acts on the loose connective tissue under the skin and performs sweeping motions.^[19]^ For deep and superficial abnormal muscles, it can relieve muscle tension, increase muscle elasticity and improve muscle blood supply.^[20]^

Reperfusion activity (RA) refers to the continuous and repetitive contraction and diastole of local muscles and related joints through the patient’s force or external force, giving intermittent vascular compression, regulating local circulation, strengthening blood flow dynamics, and promoting the recovery of ischemic tissues to normal, which is an important method to assist FSN treatment, and can further improve the effect of FSN treatment.^[21]^ The ipsilateral head-turning resistance and head-up resistance can make the left sternocleidomastoid muscle, obliquus muscle, and supraspinatus muscle continuously contract and diastole, better regulate the intramuscular blood circulation, and alleviate the muscle stiffness and contracture state.^[22]^

Therefore, we believe that the possible mechanism of FSN in the treatment of SSHL with tinnitus is: by relieving the tense and stiff muscles of the neck, improving the blood supply of the vertebral arteries, and eventually improving the blood supply of the inner ear.

### 4.3. Summary

This case shows that the FSN has good efficacy in the treatment of SSHL with tinnitus. By improving the condition of the relevant neck muscles and the blood supply of the vertebrobasilar artery, it can improve the patient’s hearing and reduce the loudness and duration of tinnitus. The results of this report showed that the patient’s high-frequency hearing was not recovered satisfactorily, and only the low-frequency hearing was improved more significantly. This is a case report, and a high-quality, large-sample, multicentre randomized controlled trial is needed to evaluate the efficacy of FSN in the treatment of SSHL with tinnitus.

## Author contributions

**Conceptualization:** Jin Lu.

**Funding acquisition:** Jin Lu.

**Visualization:** Yue Wang, Xinghao Ding.

**Writing – original draft:** Jiarui Zhang.

**Writing – review & editing:** Jin Lu.
